# The short medication adherence scale (SMAS-7): Development and psychometric validation in a general population sample

**DOI:** 10.1016/j.rcsop.2025.100676

**Published:** 2025-10-25

**Authors:** Fouad Sakr, Mariam Dabbous, Jihan Safwan, Mohamad Rahal, Pascale Salameh

**Affiliations:** a School of Pharmacy, Lebanese International University, Beirut, Lebanon; b INSPECT-LB: Institut National de Santé Publique, d'Épidémiologie Clinique et de Toxicologie-Liban, Beirut, Lebanon; c Inserm U1094, IRD UMR270, Univ. Limoges, CHU Limoges, EpiMaCT - Epidemiology of chronic diseases in tropical zone, Institute of Epidemiology and Global Health – Michel Dumas, OmegaHealth, Limoges, France; d Aging Research Team, Center for Epidemiology and Research in POPulation health (CERPOP), Université de Toulouse (formerly UPS – Toulouse III), Inserm, 31000 Toulouse, France; e Université de Toulouse (formerly UPS – Toulouse III), 31062 Toulouse, France; f Faculty of Public Health, Lebanese University, Fanar, Lebanon; g Faculty of Pharmacy, Lebanese University, Beirut, Lebanon; h Gilbert and Rose-Marie Chagoury School of Medicine, Lebanese American University, Byblos, Lebanon; i Department of Primary Care and Population Health, University of Nicosia Medical School, Nicosia, Cyprus

**Keywords:** Medication adherence, Scale validation, Psychometrics, SMAS-7, Chronic and acute treatment

## Abstract

**Background:**

Medication adherence is essential for treatment success across both chronic and acute conditions. However, concise, multidimensional, and broadly applicable validated tools to measure medication adherence remain scarce.

**Objectives:**

This study primarily aimed to develop and validate the Short Medication Adherence Scale (SMAS-7) and, secondarily, to assess adherence levels and related factors in a general adult population.

**Methods:**

A cross-sectional study was conducted among Lebanese adults aged ≥18 years from the general population. Individuals who declined follow-up contact for the test-retest phase were excluded. The 7-item SMAS-7, developed from the LMAS-14, was administered electronically in Arabic using an online questionnaire. Exploratory and confirmatory factor analyses (EFA and CFA) were performed on two random subsamples to assess factorial structure and model fit. Internal consistency (Cronbach's α, McDonald's ω), test-retest reliability (ICC), and construct and criterion validity were evaluated. Multivariable logistic regression identified predictors of adherence using a ROC curve-derived SMAS-7 cut-off score.

**Results:**

A total of 501 participants were included in the study. EFA revealed a 3-factor structure, psychological, economic, and behavioral domains, supported by KMO = 0.830 and significant Bartlett's test (*P* < 0.001). CFA confirmed the structure with excellent fit (χ^2^/df = 1.757, CFI = 0.992, TLI = 0.985, RMSEA = 0.055, SRMR = 0.020). The SMAS-7 demonstrated high internal consistency (α = 0.889, ω = 0.945) and test-retest reliability (ICC = 0.779). Criterion validity was excellent (AUC = 0.985; sensitivity = 91.2 %; specificity = 96.0 %). Suboptimal adherence was observed in 52.3 % of participants. Significant predictors of adherence included gender (*P* = 0.030), region (*P* = 0.014), financial well-being (*P* = 0.002), chronic illness (*P* = 0.009), communication barriers (*P* = 0.013), and patient perception (*P* = 0.029).

**Conclusion:**

The SMAS-7 demonstrated strong preliminary psychometric properties in this initial validation study. It offers a valuable resource for researchers, clinicians, and policymakers seeking to monitor and enhance adherence behaviors. While these findings are encouraging, further studies in diverse populations and clinical settings are required to confirm its external validity and generalizability. The findings revealed suboptimal adherence and underscored the multifaceted nature of its predictors, highlighting the need for targeted, multidimensional interventions.

## Highlights

• A 7-item scale (SMAS-7) was developed to assess medication adherence.
• The scale showed a 3-factor structure: psychological, economic, and forgetfulness.
• CFA confirmed excellent model fit for the SMAS-7 structure.
• SMAS-7 demonstrated strong reliability and criterion validity.
• Suboptimal medication adherence was linked to clinical, social, and economic factors.

## 1 Introduction

Medication adherence refers to the extent to which patients take their medications as prescribed or recommended.^1^ It is essential for achieving optimal therapeutic outcomes in both chronic and acute conditions.^2^ However, non-adherence remains a widespread issue.^3^ For instance, nearly 50 % of patients with chronic diseases in developed countries do not follow their prescribed long-term treatment plans, leading to poor health outcomes and increased healthcare costs.^4^^,^^5^ Accurately measuring medication adherence is particularly challenging. While objective methods such as pill counts or pharmacy refill records are reliable, they can be resource-intensive.^6^^,^^7^ In contrast, self-report tools, such as questionnaires or assessment scales, are more practical and feasible for large-scale use, though they are inherently subjective. Therefore, rigorous validation is essential to ensure accurate measurement and reduce bias.^8^^,^^9^

Studies on medication adherence have consistently identified sociodemographic (e.g., age, gender, income, education), economic (e.g., insurance coverage, medication cost), and clinical factors (e.g., disease burden, polypharmacy) as strong predictors of patient behavior.^10^, ^11^, 12., 13. However, an important gap in the literature is that most adherence research focuses primarily on patients with chronic conditions and long-term medication regimens. It is equally important to assess adherence among nonchronic patients, such as those prescribed antimicrobials or other short-term treatments, as poor adherence in these contexts may lead to treatment failure and increased rates of complications.^14^^,^^15^ Moreover, patient experiences throughout the healthcare journey also play a pivotal role in shaping adherence. For example, community pharmacists are often the final point of contact for prescription dispensing, medication refills, and over-the-counter recommendations. As such, the patient experience within the community pharmacy setting may significantly influence adherence behavior.^16^^,^^17^ While numerous qualitative and patient-centered studies have explored how real-world experiences influence medication-taking behavior,^18^, ^19^, 20., 21, 22 fewer efforts have integrated these experiential dimensions into quantitative adherence measurement tools. This underscores the need for multidimensional instruments that capture the psychological, economic, and experiential aspects of adherence within diverse real-world contexts.

In Lebanon, socioeconomic pressures, healthcare system fragmentation, and cultural factors further complicate the medication adherence construct.^23^ The Lebanese Medication Adherence Scale (LMAS-14) was developed and validated to capture this multidimensional complexity, encompassing economic constraints, psychological, and occupational factors, and has demonstrated good psychometric properties in various populations, including patients with hypertension,^24^ diabetes,^25^ hypothyroidism,^26^ and stroke.^27^ However, its 14-item length may limit its routine use in busy clinical or research settings.

Shorter adherence measuring tools, such as 3-item scales, have demonstrated validity and ease of use. Nonetheless, although concise and practical, these very short-form scales frequently lack the dimensional breadth needed to comprehensively capture the multifaceted nature of medication adherence.^28^^,^^29^ Furthermore, many are disease-specific or population-targeted. For instance, the 7-item Diabetes Medication Adherence Scale (DMAS-7), which strongly correlates with the LMAS-14 and effectively predicts glycemic control, is a promising tool.^30^ Yet, it is narrowly focused on diabetic populations and cannot be generalized to broader contexts involving other chronic or acute conditions.

Combining acute and chronic medication contexts within a single conceptual framework is important for developing a generalizable adherence measure. Although clinical indications and treatment durations differ, many behavioral and economic determinants of adherence such as forgetfulness, intentional discontinuation when symptoms resolve, or cost-related barriers are shared across settings. Reviews show that non-adherence is influenced by patient-, therapy-, condition- and system-related factors, and that much research focuses on chronic therapies while short-term treatment adherence is understudied.^31^ Social determinants of health have been shown to affect adherence even when the therapy duration or disease type varies.^32^ For these reasons, including participants from a general adult population provides an inclusive and pragmatic foundation for developing a multidimensional scale applicable to individuals regardless of diagnosis or therapy duration.

Although numerous self-reported instruments have been developed to assess medication adherence, most existing scales are disease-specific, lengthy, or context-restricted, limiting their general applicability in community or low-resource settings. Systematic reviews have highlighted that many tools show inconsistent measurement properties across populations and limited validation outside chronic-disease contexts.33., 34., 35, 36, 37. These limitations underscore the need for concise, multidimensional, and culturally adaptable tools capable of assessing adherence among individuals using either chronic or acute therapies.

Although medication use patterns differ between acute and chronic conditions, common behavioral mechanisms underlie adherence across both contexts. Patients may forget doses, discontinue treatment prematurely upon symptom improvement, or face financial and access barriers regardless of treatment duration. Therefore, a common tool was designed to capture these shared determinants through a brief, multidimensional framework. It is intended as a general screening and research tool rather than a disease-specific instrument, providing a practical approach for assessing adherence among individuals using both short-term and long-term therapies. Validated tools capable of assessing medication adherence across both chronic and acute treatment contexts in the general population are still lacking. To address this gap, the primary objective of this study was to develop and validate the 7-item Short Medication Adherence Scale (SMAS-7), a streamlined tool designed for broader applicability. In addition, the study aimed to evaluate adherence levels among Lebanese adults and identify potential predictors, including sociodemographic, economic, clinical, and patient experience factors. While a concise 7-item format cannot fully capture the multifaceted nature of medication adherence, the SMAS-7 was designed to offer a pragmatic balance between brevity and multidimensionality. By preserving core psychological, economic, and behavioral dimensions within a short self-report format, it aims to enhance feasibility in both research and practice, particularly in resource-limited or high-throughput settings. Therefore, rather than serving as a comprehensive solution, the SMAS-7 should be viewed as a practical first-step or “stepping stone” tool that can complement or precede more detailed adherence assessments in future work.

## 2 Methods

### 2.1 Development of the 7-item short medication adherence scale (SMAS-7)

A panel of five experts, all co-authors of the study, conducted the item selection process using a Modified Delphi technique. Consensus was defined as ≥80 % agreement and was achieved over three rounds of iterative rating and feedback. The expert panel included four pharmacists (two pharmacoepidemiologists and two pharmacotherapy specialists who also serve as community pharmacy preceptors) and one pharmacologist. To mitigate potential bias arising from author involvement, each expert independently rated item relevance, clarity, and conceptual coverage according to predefined criteria. During each Delphi round, experts independently rated the relevance, clarity, and conceptual distinctiveness of each LMAS-14 item using a 4-point Likert scale (1 = not relevant to 4 = highly relevant). In Round 1, all 14 original items were reviewed for potential retention or modification. Items with ≥80 % agreement for high relevance were retained, while those below the threshold were either reworded or reconsidered in subsequent rounds. In Round 2, experts re-evaluated modified items and proposed new items to fill conceptual gaps. Round 3 served to confirm consensus on the final set of retained and newly added items. Throughout all rounds, anonymous feedback summaries were shared to allow for iterative refinement without direct group influence, ensuring methodological rigor and minimizing bias.

The SMAS-7 was developed from the original 14-item LMAS-14 through systematic item selection and the introduction of one new item. The aim was to develop a concise yet conceptually comprehensive scale that preserves the multidimensional construct of the LMAS-14. Items were carefully selected to ensure broad, generalizable assessment of medication adherence while avoiding redundancy and maintaining clarity and relevance. One item was selected to assess general forgetfulness in taking medications, and another to reflect delays in refilling medication packs. Two items were selected to address discontinuation of medication without consultation, based on either improvement in laboratory results or lack of perceived clinical benefit. Two additional items were retained to reflect financial barriers, including lack of insurance coverage and high medication cost. One new item was introduced to capture premature discontinuation upon feeling better, thereby addressing a conceptual gap not covered by the selected items.

The resulting seven items yielded a concise and comprehensive scale that is projected to be usable for both chronic and acute medication use assessment, and suitable and practical for clinical practice and research. The SMAS-7 was administered in Arabic. Since the original LMAS-14 was developed and validated in Arabic, the retained items required only minor linguistic adjustments. The newly introduced item was drafted in Arabic, back-translated into English by a bilingual expert, and reviewed for conceptual equivalence by the research team. All items are measured using a 4-point scale ranging from 1 (lower adherence) to 4 (greater adherence). The total SMAS-7 score is computed by summing all item responses, with higher scores indicating better medication adherence.

### 2.2 Study design and participants

This was a cross-sectional study that took place between February and May 2025. Participants were recruited using a snowball sampling approach. The research team initially disseminated the online survey link through professional, academic, and personal networks via widely used social media platforms (WhatsApp, Facebook, Instagram, and LinkedIn). Recipients were encouraged to forward the survey link to other eligible individuals within their networks, thereby expanding the reach of the sample across all Lebanese districts. Participation was voluntary, and the survey's introductory page described the study purpose, estimated duration, confidentiality safeguards, and the right to withdraw at any time. Participants were required to review an electronic informed consent statement and agree to participate before being granted access to the questionnaire. Eligible participants were Lebanese adults aged 18 years or older, able to read and understand Arabic, and willing to provide electronic informed consent and participate in both the baseline and follow-up (retest) phases. Individuals who declined follow-up contact or submitted incomplete questionnaires were excluded.

The introductory section of the questionnaire outlined the study's objectives and estimated duration, and emphasized the voluntary nature of participation, including the right to withdraw at any time. Participants were informed that providing a contact number or email address was required for follow-up, as they would be contacted later to complete a second round of assessment for test-retest reliability. Individuals who declined future contact were excluded from the study.

The questionnaire was administered in Arabic, the native and widely understood language in Lebanon. A pilot study involving 10 participants was conducted to assess the clarity and comprehensibility of the instrument. These individuals were recruited through convenience sampling from the research team's social and professional networks and met the same inclusion criteria as the main study (Lebanese adults aged ≥18 years). After completing the questionnaire, participants provided written feedback through a short debriefing form, indicating any items that were unclear, confusing, or difficult to interpret. The research team reviewed this feedback and made minor wording adjustments accordingly. Data from the pilot phase were excluded from the final analysis. The initial recruitment and data collection phase occurred from February to March 2025, while follow-up contact was made in May 2025 to facilitate the test-retest evaluation. All participants included in the baseline assessment were successfully re-contacted for the retest phase, as individuals who declined future follow-up were excluded during initial recruitment. This design minimized attrition and ensured that the same sample contributed to both the initial and retest datasets used for assessing test–retest reliability. Email addresses or phone numbers were used as unique identifiers to match and combine responses from the initial and retest phases. These identifiers were not stored in the analysis dataset, thereby ensuring the anonymity and confidentiality of all participants. The final questionnaire included approximately 125 items and required an average of 15 min to complete online. The full Arabic version of the questionnaire is provided in Appendix 1.

### 2.3 Measures and variables

The study questionnaire was structured into six sections. The first section collected sociodemographic and socioeconomic data, including age, gender, region, education level, employment status, and household income. Financial distress and well-being were assessed using the InCharge Financial Distress/Financial Well-being (IFDFW) scale, a validated 8-item subjective measure. Each item is rated on a 1 to 10 scale, with higher scores indicating better financial well-being.^38^ In the current sample, the IFDFW scale had a Cronbach's α of 0.960.

The second section captured clinical characteristics, including health status, the presence and type of chronic diseases or comorbidities, chronic medication use (type and quantity), ease of access to healthcare, and insurance or health coverage. This section also included the LMAS-14, which demonstrated a Cronbach's α of 0.934 in this study.

The third section assessed patient-reported experiences related to acquiring medications, receiving counseling, and interacting with community pharmacists. This focus reflects the Lebanese context, where community pharmacies serve as the primary point of medication access, whether by prescription refill or pharmacist-guided non-prescription recommendation.^39^ This section utilized the Patient-Pharmacist Relationship Measurement Tool, validated in Lebanon, comprising three indices: the Patient Expectation Index (11 items), the Barriers to Communication with Pharmacist Index (7 items), and the Patient Perception Index (14 items).^40^ Index scores are calculated by summing item responses, with higher scores reflecting higher levels of expectations, barriers, and perceptions, respectively. Cronbach's α coefficients were 0.745 for the Expectation Index, and 0.905 for both the Barriers and Perception Indices.

The fourth section explored additional aspects of patient experience and satisfaction using a modified version of the Patient Satisfaction Questionnaire Short Form (MA-PSQ18). This 18-item validated instrument measures satisfaction with pharmacists and community pharmacies across seven subscales: general satisfaction, technical quality, interpersonal manner, time spent with the pharmacist, and accessibility/convenience.^41^ Subscale and total scores are generated by summing item responses, with higher scores indicating greater satisfaction. In the current sample, the MA-PSQ18 showed a Cronbach's α of 0.952.

The fifth section included the newly developed SMAS-7, with its 7 items and scoring method described earlier.

The sixth section incorporated the EQ-5D-5L to assess overall health status and quality of life. This standardized instrument includes two components: (1) the EQ-5D descriptive system, which covers five dimensions (mobility, self-care, usual activities, pain/discomfort, and anxiety/depression), each scored on five levels (1 = no problems to 5 = extreme problems), and (2) the EQ Visual Analogue Scale (EQ VAS), where respondents rate their health from 0 (worst imaginable) to 100 (best imaginable).^42^

In the retest phase of this study, the fourth and fifth sections (MA-PSQ18 and SMAS-7) were re-administered to assess test-retest reliability. The SMAS-7 was retested as part of the current study, while the MA-PSQ18 was included for reliability assessment in a future investigation within the same research project.

### 2.4 Ethical aspects

The study protocol was approved by the Ethics and Research Committee of the School of Pharmacy at the Lebanese International University (Protocol No. 2025ERC-009-LIUSOP). Informed consent was obtained from all participants prior to their enrollment. The study was conducted in accordance with the ethical principles outlined in the Declaration of Helsinki, and participant confidentiality was upheld at all stages of the study.

### 2.5 Sample size calculation

The minimum required sample size was determined using a two-step approach. In the first step, the calculation was performed using G*Power version 3.1.9.7, based on a logistic regression Wald test. A two-tailed test was specified with an alpha level of 0.05, power of 0.80, and an assumed medium effect size (odds ratio ≈ 1.5). The expected adherence probability was set at 49.8 %, based on previously published literature.^23^ This calculation indicated that 208 participants would be sufficient to detect the expected effect size. In the second step, to ensure model stability for the planned multivariable analysis, the sample size was adjusted using the Events Per Variable (EPV) method, which recommends at least 10 outcome events per predictor.^43^ The final required sample size was calculated using the formula^44^:

$$N=\frac{\mathrm{EPV}\times P}{\mathrm{Pr}\left(Y=1\right)}$$

where *N* is the sample size, *P* is the number of predictors, and Pr(*Y = 1*) is the expected event rate. Allowing 20 predictors in the model, an EPV of 10, and an adherence rate of 49.8 %, the minimum required sample size was calculated as:

$$\frac{10\times 20}{0.498}\approx 402\;\mathrm{participants}$$

For the scale validation, a participant-to-item ratio of 10:1 was applied,^45^ requiring a minimum of 70 participants for the seven-item SMAS-7 scale. Since two independent validations were planned, a total of 140 participants was considered necessary for this purpose. Accordingly, the final minimum sample size of 402 participants was required to meet all study objectives, including scale validation and multivariable analysis, with 80 % statistical power, a 95 % confidence level, and a 5 % margin of error.

### 2.6 Statistical analysis

Data were analyzed using R version 4.5.0 (R Foundation for Statistical Computing, Vienna, Austria) and RStudio version 2025.05.0 + 496 (Mariposa Orchid, RStudio, PBC). The electronic survey form required participants to respond to all items before submission; therefore, no missing or incomplete responses were present in the dataset, and all participants were included in the analyses. Descriptive statistics were used to summarize participants' sociodemographic, socioeconomic and clinical characteristics, and patient-reported experiences. Continuous variables were reported as means (± standard deviations, SD), and categorical variables as frequencies and percentages.

The total sample was randomly divided in R into two equal subsamples (Sample 1 and Sample 2). Exploratory factor analysis (EFA) was conducted on Sample 1 using the *psych* and *GPArotation* packages, with Promax rotation applied due to the expected correlation among the SMAS-7 items. Sampling adequacy was assessed using the Kaiser-Meyer-Olkin (KMO) test and Bartlett's test of sphericity. Factors with Eigenvalues greater than 1 were retained. Confirmatory factor analysis (CFA) was then performed on Sample 2 using the *lavaan* package with maximum likelihood estimation to validate the factor structure identified in the EFA. Model fit was evaluated using the following indices: chi-square per degree of freedom (χ^2^/df), Comparative Fit Index (CFI), Tucker-Lewis Index (TLI), Root Mean Square Error of Approximation (RMSEA), and Standardized Root Mean Square Residual (SRMR). Values of χ^2^/df < 3, CFI and TLI ≥ 0.95, RMSEA ≤0.06 (≤ 0.08 acceptable), and SRMR ≤0.08 were considered indicative of good model fit.^46^^,^^47^ IBM SPSS Amos version 24.0 (IBM Corp., Armonk, NY, USA) was used to visualize the CFA path diagram and to report standardized factor loadings and correlations between latent factors.

All remaining analyses were performed on the total sample. Internal consistency was initially assessed using Pearson correlation coefficients (r) between the SMAS-7 total score, subscale scores, and individual items. Additional internal consistency and reliability estimates were obtained using Cronbach's α and McDonald's ω, computed via the *psych* and *semTools* packages, respectively. McDonald's ω was calculated only for the total scale and for subscales containing three or more items, as coefficients based on fewer than three items are generally considered statistically unstable and theoretically unreliable for representing a latent construct. Test-retest reliability was evaluated using intraclass correlation coefficients (ICCs) with 95 % confidence interval (95 % CI), using the *psych* package.

Construct validity was assessed through convergent, concurrent, and divergent validity testing using Pearson correlation coefficients. Convergent validity was evaluated through correlation with the LMAS-14; concurrent validity was assessed via correlations with the IFDFW, Patient Perception Index, and MA-PSQ18; and divergent validity was tested using the EQ VAS. Criterion validity was assessed using receiver operating characteristic (ROC) curve analysis, conducted with the *pROC* and *ggplot2* packages. The LMAS-14 was used as the reference standard, with a cut-off score of 38 to distinguish between better and lower adherence, based on thresholds established in previous literature.^24^ Sensitivity and specificity at the optimal SMAS-7 cut-off point were determined using Youden's J index.

The SMAS-7 score was dichotomized at the ROC-derived cut-off point to classify participants into lower and better adherence groups. Univariate logistic regression analyses were conducted to examine associations between medication adherence and sociodemographic, socioeconomic, clinical, and patient-reported experience variables. Variables with a *P* value <0.20 in the univariate analysis were included in two multivariable logistic regression models, using the Backward Likelihood Ratio method. Model 1 included sociodemographic and socioeconomic variables, and Model 2 included clinical and patient-reported experience variables. A third model (Model 3) combined all variables that were statistically significant in the first two models. Model fit and adequacy were evaluated using the Hosmer–Lemeshow goodness-of-fit test, and Nagelkerke R^2^ to assess the proportion of explained variance. Adjusted odds ratios (aORs), 95 % CI, and *P* values were reported. A P value <0.05 was considered statistically significant.

## 3 Results

### 3.1 Sociodemographic and socioeconomic characteristics

A total of 501 participants were included in the study. The mean age was 34.66 years (±15.17). The majority of participants were female (67.27 %) and resided predominantly in South Lebanon (68.46 %), followed by Beirut (22.95 %). More than half were single (52.30 %), and the vast majority had attained a university-level education (81.64 %). Regarding employment status, 56.49 % were employed or self-employed, while 40.32 % were unemployed. In terms of monthly income, approximately one-third of participants reported a household income of more than 1500 USD (31.94 %), while 21.96 % reported earning less than 500 USD. The mean IFDFW score was 47.94 (±19.92). The detailed sociodemographic and socioeconomic characteristics of the participants are presented in Supplementary Table 1 (Appendix 2).

### 3.2 Clinical characteristics and patient-reported experiences

Around one-quarter of participants reported having a chronic illness (25.95 %). On average, participants had 1.33 comorbidities (±1.56) and took 1.04 chronic daily medications (±1.66). The majority reported easy access to healthcare (79.84 %), and 61.68 % had some form of health coverage, most commonly private insurance or the National Social Security Fund. Regarding pharmacist counseling, 22.95 % received it regularly, and 47.11 % reported counseling sessions lasting 5–10 min. Mean scores for patient-reported experiences were 20.09 (±2.01) for the Patient Expectation Index, 9.69 (±2.70) for the Barriers to Communication score, 35.22 (±6.51) for the Patient Perception Index, and 69.35 (±10.75) for the MA-PSQ18. The mean score for the LMAS-14 was 36.66 (±11.04), while the mean EQ VAS score was 76.16 (±20.82). The detailed clinical characteristics and patient-reported experiences are presented in Supplementary Table 2 (Appendix 3).

### 3.3 Validation of the SMAS-7

#### 3.3.1 Exploratory factor analysis

An EFA was conducted on Sample 1 (*N* = 250) to examine the underlying structure of the SMAS-7. All seven items were retained and extracted using Promax rotation. No items showed excessive intercorrelation (*r* > 0.9), low factor loadings (< 0.30), or low communalities (< 0.30). The KMO measure of sampling adequacy was 0.830, and Bartlett's test of sphericity was significant (*P* < 0.001), indicating suitability for factor analysis.

The analysis yielded a 3-factor solution with Eigenvalues greater than 1, accounting for 85.26 % of the total variance. Factor 1 comprised three items reflecting psychological factors, Factor 2 included two items related to economic factors, and Factor 3 consisted of two items associated with behavioral factor. The Promax rotated component matrix is presented in Table 1.

**Table 1:** Promax rotated component matrix of the SMAS-7 in Sample 1.

SMAS-7 item #	SMAS-7 items	Factor 1	Factor 2	Factor 3	Communalities
SMAS3	Do you stop taking your medication without consulting your doctor if the laboratory tests show improvement during treatment period?	0.998			0.900
SMAS5	Do you stop taking your medication without consulting your doctor if you feel better during treatment period?	0.914			0.865
SMAS4	Do you stop taking your medication without consulting your doctor if you do not feel better during treatment period?	0.803			0.798
SMAS6	Do you stop taking your medication if your insurance does not cover it?		0.955		0.901
SMAS7	Will you stop buying your medication packs if you considered them expensive?		0.933		0.896
SMAS2	Do you get late when it comes to buying your medication packs when they become empty?			0.914	0.812
SMAS1	Do you forget to take your medication?			0.878	0.797
	*Percentage of variance explained*	35.94 %	26.27 %	23.06 %	

Factor 1 = Psychological; Factor 2 = Economic; Factor 3 = Behavioral.

Total percentage of variance explained: 85.26 %.

Kaiser-Meyer-Olkin (KMO) = 0.830.

Bartlett's test of sphericity: P < 0.001.

#### 3.3.2 Confirmatory factor analysis

A CFA was conducted on Sample 2 (*N* = 251) to validate the 3-factor structure of the SMAS-7 identified through the EFA in Sample 1. The maximum likelihood estimation method indicated that the overall model fit was good, with χ^2^/df = 19.330/11 = 1.757 and a non-significant *p*-value (*P* = 0.057), suggesting that the hypothesized model did not significantly deviate from the observed data.

**Fig. 1:**
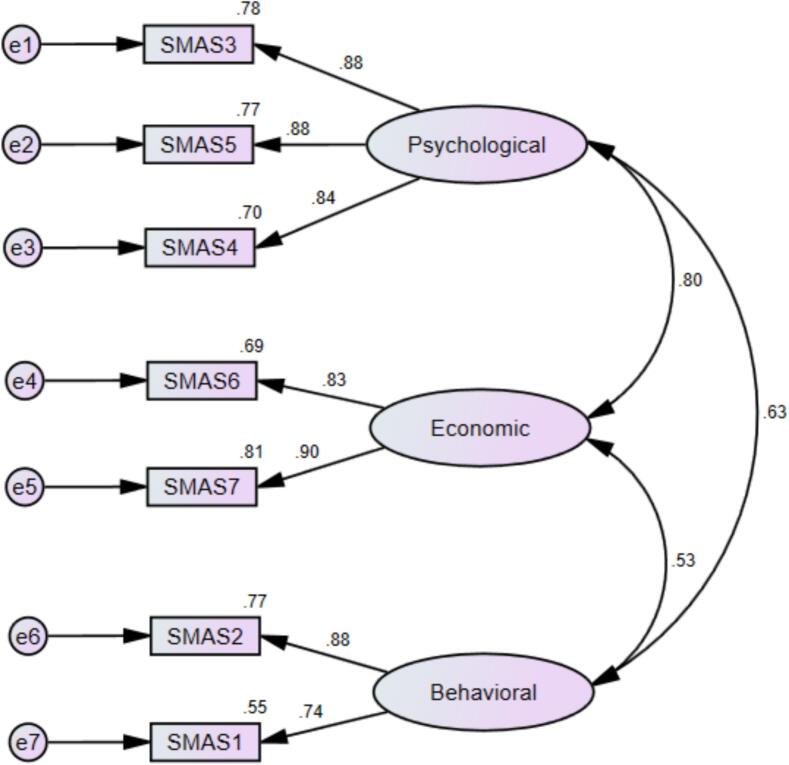
Standardized estimates of factor loadings from the confirmatory factor analysis (CFA) of the SMAS-7 items in sample 2. e = error term.

Model fit was further supported by additional indices. The CFI was 0.992 and the TLI was 0.985, both exceeding the recommended threshold of 0.95. The RMSEA was 0.055 (90 % CI: 0.001–0.094), with a close-fit test p-value of 0.378, indicating good model fit. The SRMR was 0.020, well below the recommended cut-off of 0.08. Fig. 1 illustrates the standardized factor loadings and structural paths of the SMAS-7 CFA model.

#### 3.3.3 Internal consistency and reliability

Table 2 presents the Pearson correlation matrix for the SMAS-7 total scale, its three factor subscales, and individual items (in the total sample). All correlations were statistically significant at *P* < 0.001, indicating strong internal consistency across the scale. The subscales showed strong positive correlations with the total score (Factor 1: *r* = 0.913; Factor 2: *r* = 0.832; Factor 3: *r* = 0.747), supporting a coherent multidimensional structure of the SMAS-7. Items within each factor demonstrated high correlations with their corresponding subscale (ranging from *r* = 0.901 to 0.942), further confirming the internal coherence of the factors.

**Table 2:** Pearson correlation matrix of the SMAS-7 items, subscales, and total scale.

	SMAS-7 scale	Factor 1	Factor 2	Factor 3	SMAS3	SMAS5	SMAS4	SMAS6	SMAS7	SMAS1
**Factor 1**	0.913⁎	1								
**Factor 2**	0.832⁎	0.668⁎	1							
**Factor 3**	0.747⁎	0.521⁎	0.436⁎	1						
**SMAS3**	0.836⁎	0.932⁎	0.600⁎	0.464⁎	1					
**SMAS5**	0.846⁎	0.920⁎	0.630⁎	0.483⁎	0.807⁎	1				
**SMAS4**	0.831⁎	0.901⁎	0.610⁎	0.487⁎	0.755⁎	0.727⁎	1			
**SMAS6**	0.773⁎	0.612⁎	0.942⁎	0.406⁎	0.559⁎	0.575⁎	0.550⁎	1		
**SMAS7**	0.793⁎	0.647⁎	0.941⁎	0.415⁎	0.572⁎	0.610⁎	0.599⁎	0.773⁎	1	
**SMAS1**	0.666⁎	0.446⁎	0.406⁎	0.901⁎	0.388⁎	0.425⁎	0.416⁎	0.391⁎	0.374⁎	1
**SMAS2**	0.680⁎	0.492⁎	0.380⁎	0.902⁎	0.448⁎	0.446⁎	0.461⁎	0.341⁎	0.374⁎	0.624⁎

SMAS = Short Medication Adherence Scale; Factor 1 = Psychological; Factor 2 = Economic; Factor 3 = Behavioral.

⁎ *P* < 0.001.

Table 3 presents the reliability estimates for the SMAS-7 total scale and its three subscales. Cronbach's α for the SMAS-7 was 0.889, with subscale values ranging from 0.769 to 0.906. McDonald's ω was 0.945 for the SMAS-7 and 0.907 for the Factor 1 subscale; it was not estimated for Factors 2 and 3 due to having fewer than three items. Test-retest reliability, assessed using ICCs, demonstrated good temporal stability for the SMAS-7 (ICC = 0.779; 95 % CI: 0.737–0.815; P < 0.001) and for each subscale, with ICC values ranging from 0.721 to 0.740 and all *P* values <0.001.

**Table 3:** Reliability estimates for the SMAS-7 scale and subscales including internal consistency and test-retest metrics.

Scale / Subscale	Internal consistency reliability		Test-retest reliability			
	Cronbach's α	McDonald's ω	ICC	Lower 95 % CI	Upper 95 % CI	P value
SMAS-7	0.889	0.945	0.779	0.737	0.815	< 0.001
Factor 1	0.906	0.907	0.727	0.674	0.771	< 0.001
Factor 2	0.872	NA	0.740	0.690	0.782	< 0.001
Factor 3	0.769	NA	0.721	0.667	0.766	< 0.001

SMAS-7 = 7-item Short Medication Adherence Scale; Factor 1 = Psychological; Factor 2 = Economic; Factor 3 = Behavioral; ICC = intraclass correlation coefficient; CI = confidence interval; NA = not applicable (McDonald's ω not estimated for factors with fewer than 3 items).

#### 3.3.4 Convergent, concurrent, and divergent validity

Convergent validity was demonstrated by a strong, significant correlation between the SMAS-7 and LMAS-14 scores (*r* = 0.974, P < 0.001). Concurrent validity was evidenced by significant correlations between the SMAS-7 score and the IFDFW score (*r* = 0.139, *P* = 0.002), the Patient Perception Index (*r* = 0.117, *P* = 0.009), and the MA-PSQ18 score (*r* = 0.089, *P* = 0.047). Divergent validity was supported by a nonsignificant correlation between the SMAS-7 score and the EQ VAS score (*r* = 0.072, *P* = 0.108). Detailed bivariate correlations among these measures are provided in Supplementary Table 3 (Appendix 4).

#### 3.3.5 Criterion validity

**Fig. 2:**
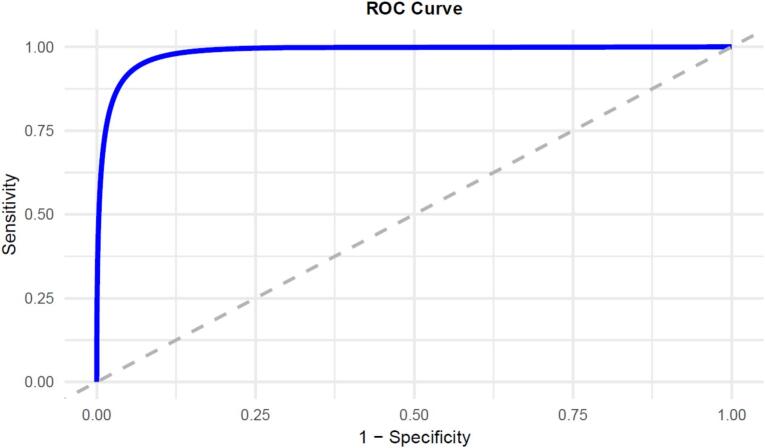
ROC curve of the SMAS-7. Participants with better medication adherence on the LMAS-14 were analyzed. Area under the curve = 0.985; 95 % CI: 0.977–0.993 (*P* < 0.001). At value = 19.50, Se = 91.24 % and Sp = 96.00 %.

The SMAS-7 had a mean score of 18.64 (±5.78), with higher scores indicating better medication adherence. ROC curve analysis was performed to assess the criterion validity of the SMAS-7 by comparing participants classified as having better versus lower adherence based on the LMAS-14. The analysis identified an optimal cut-off score of 19.50 on the SMAS-7 for determining better adherence. This cut-off yielded a sensitivity of 91.24 % and a specificity of 96.00 %, with an area under the curve (AUC) of 0.985 (95 % CI: 0.977–0.993; *P* < 0.001). The ROC curve for the SMAS-7 is shown in Fig. 2.

### 3.4 Assessment of medication adherence and its associated factors

The SMAS-7 score was dichotomized at the cut-off point of 19.50, as determined by ROC curve analysis, to classify participants into lower and better medication adherence groups. Overall, 52.30 % of participants were classified as having lower adherence, while 47.70 % demonstrated better adherence. Univariate logistic regression analysis of sociodemographic and socioeconomic characteristics showed that older age was significantly associated with better adherence (OR = 1.020, *P* = 0.001). Female participants had significantly lower adherence compared to males (OR = 0.449, *P* < 0.001). Compared to residents of Beirut, those living in Mount Lebanon (OR = 0.179, *P* = 0.005) and South Lebanon (OR = 0.353, P < 0.001) had significantly lower adherence. Being married was positively associated with adherence (OR = 1.563, *P* = 0.016). Monthly income showed a strong relationship with adherence: participants earning 1000–1500 USD (OR = 1.969, *P* = 0.012) or more than 1500 USD (OR = 2.694, P < 0.001) reported significantly better adherence. Additionally, higher scores on the IFDFW scale, reflecting better financial well-being, were associated with improved adherence (OR = 1.019, P < 0.001).

Several clinical and patient-reported experience variables were also significantly associated with medication adherence. Participants with chronic illness had better adherence than those without (OR = 2.329, P < 0.001). Having health coverage through the National Social Security Fund (OR = 2.026, *P* = 0.008) or private insurance (OR = 1.977, *P* = 0.001) was significantly associated with better adherence compared to having no coverage. Among patient-reported experiences, higher scores on the Patient Perception Index (OR = 1.060, P < 0.001) and the MA-PSQ18 (OR = 1.027, *P* = 0.002) were significantly associated with better adherence, while higher scores on the Barriers to Communication with Pharmacist Index (OR = 0.884, P < 0.001) were associated with lower adherence. The detailed results of the univariate logistic regression analyses are presented in Supplementary Table 4 (Appendix 5).

#### 3.4.1 Multivariable analysis

Table 4 presents the results of three models of multivariable logistic regression analyses conducted to identify predictors of medication adherence, using the dichotomized SMAS-7 score as the dependent variable. In Model 1, which included sociodemographic and socioeconomic variables, female participants had significantly lower adherence compared to males (aOR = 0.594, *P* = 0.014). Residence in Mount Lebanon (aOR = 0.276, *P* = 0.045) and South Lebanon (aOR = 0.499, *P* = 0.004) was associated with lower adherence compared to Beirut. In contrast, better financial well-being, as measured by the IFDFW score, was significantly associated with higher adherence (aOR = 1.017, P = 0.001).

Model 2 included clinical and patient-reported experience variables. Participants with chronic illness showed better adherence than those without (aOR = 1.881, P = 0.004). Having health coverage through the National Social Security Fund (aOR = 1.808, *P* = 0.032) or private insurance (aOR = 1.595, *P* = 0.038) was significantly associated with greater adherence compared to having no coverage. Additionally, higher scores on the Patient Perception Index (aOR = 1.047, P = 0.002) were associated with better adherence, while higher scores on the Barriers to Communication with Pharmacist Index (aOR = 0.911, P = 0.008) were associated with lower adherence.

In the final model (Model 3), which included all variables found to be significant in Models 1 and 2, female gender (aOR = 0.628, *P* = 0.030), residence in South Lebanon (aOR = 0.543, P = 0.014), better financial well-being (aOR = 1.016, P = 0.002), chronic illness (aOR = 1.827, *P* = 0.009), higher communication barriers (aOR = 0.914, *P* = 0.013), and higher patient perception (aOR = 1.034, *P* = 0.029) remained significant independent predictors of medication adherence.

**Table 4:** Multivariable logistic regression analysis of predictors of medication adherence using the dichotomized SMAS-7 score as the dependent variable.

**Model 1: including sociodemographic and socioeconomic characteristics**⁎				
**Variable**	**Adjusted OR (aOR)**	**95 % CI**		**P value**
		**Lower**	**Upper**	
**Age**	1.013	1.000	1.026	0.051
**Gender**				
Female vs. Male	0.594	0.392	0.899	0.014
**Region** (reference: Beirut)				
Bekaa	2.159	0.542	10.804	0.298
Mount Lebanon	0.276	0.070	0.914	0.045
North	0.462	0.159	1.310	0.147
South	0.499	0.309	0.802	0.004
**Total IFDFW score**	1.017	1.007	1.027	0.001

**Model 2: including clinical characteristics and patient-reported experiences**⁎⁎				

**Variable**	**Adjusted OR (aOR)**	**95 % CI**		**P value**
		**Lower**	**Upper**	
**Current health status**				
Chronic illness vs No illness	1.881	1.225	2.904	0.004
**Health coverage** (reference: No coverage)				
National Social Security Fund (NSSF)	1.808	1.053	3.122	0.032
Public insurance	1.219	0.655	2.251	0.529
Private insurance	1.595	1.028	2.479	0.038
**Barriers for Communication with Pharmacist score**	0.911	0.849	0.976	0.008
**Patient Perception Index score**	1.047	1.017	1.079	0.002

**Model 3: including statistically significant sociodemographic, socioeconomic, clinical, and patient-reported experience characteristics from Model 1 and Model 2**⁎⁎⁎				

**Variable**	**Adjusted OR (aOR)**	**95 % CI**		**P value**
		**Lower**	**Upper**	
**Gender**				
Female vs. Male	0.628	0.412	0.956	0.030
**Region** (reference: Beirut)				
Bekaa	2.198	0.507	9.525	0.292
Mount Lebanon	0.318	0.088	1.154	0.081
North	0.525	0.18	1.531	0.238
South	0.543	0.333	0.883	0.014
**Total IFDFW score**	1.016	1.006	1.026	0.002
**Current health status**				
Chronic illness vs No illness	1.827	1.162	2.872	0.009
**Barriers for Communication with Pharmacist score**	0.914	0.851	0.981	0.013
**Patient Perception Index score**	1.034	1.003	1.066	0.029

OR: odds ratio; aOR: adjusted odds ratio; CI: confidence interval; IFDFW: InCharge Financial Distress/Financial Well-Being Scale.

⁎ Variables initially included in the model: age; gender; region; marital status; monthly income; total IFDFW score.

⁎⁎ Variables initially included in the model: current health status; easy access to healthcare; health coverage; Do you receive regular counseling by a pharmacist?; Barriers for Communication with Pharmacist score; Patient Perception Index score; MA-PSQ18 score.

⁎⁎⁎ Variables initially included in the model: age; gender; region; total IFDFW score; current health status; health coverage; Barriers for Communication with Pharmacist score; Patient Perception Index score.

## 4 Discussion

This study developed and psychometrically validated the SMAS-7, a concise, multidimensional instrument designed for use across both chronic and acute medication contexts in the general population. The scale showed strong psychometric performance across factorial validity, internal consistency, temporal stability, and criterion validity measures. Beyond its validation, the study also provided insight into adherence behavior in Lebanon, revealing suboptimal patterns and identifying sociodemographic, economic, clinical, and experiential determinants. The mechanisms underlying non-adherence reflected the three domains captured by the SMAS-7: psychological factors such as discontinuation after perceived recovery or symptom persistence, behavioral aspects including forgetfulness or delays in purchasing medications, and economic barriers related to cost and insurance coverage. These patterns confirm that medication-taking behavior is shaped by an interplay of personal, social, and financial determinants.

The development and validation of the SMAS-7 addressed a clear gap in the literature for concise yet conceptually robust instruments that capture the multifactorial nature of medication adherence across diverse populations. Although very brief tools, such as 3-item adherence scales, are easy to administer, they often overlook critical dimensions such as economic barriers and psychological commitment during symptom fluctuation, factors particularly relevant in low-resource and culturally nuanced contexts like Lebanon.^23^^,^48., 49., 50

The SMAS-7 was developed to preserve the multidimensional conceptual framework of comprehensive adherence tools while enhancing brevity and feasibility for use in both clinical and research settings. Psychometric analyses confirmed a coherent three-factor structure, psychological, economic, and behavioral, demonstrating the structural validity and conceptual soundness of the instrument.^24^^,^^27^ The economic factor is particularly pertinent to the Lebanese context, where high out-of-pocket expenditures and inconsistent insurance coverage continue to hinder medication continuity.^51^^,^^52^ Likewise, the psychological factor reflects self-regulatory decision-making influenced by symptom perception, a common adherence barrier across both chronic and short-term therapies.^53^

The SMAS-7 demonstrated strong internal consistency, confirming that its items measure a unified construct while capturing multiple relevant dimensions. The reliability pattern further supports a coherent multidimensional structure, as McDonald's ω typically provides a more accurate estimate of reliability when items contribute unequally to their underlying factors.^54^ Moreover, test-retest results confirmed good temporal stability, underscoring the scale's applicability in both cross-sectional and longitudinal designs. Overall, these psychometric indices exceed recommended standards for health-related measurement tools.^45^^,^^55^

Construct validity was rigorously examined through convergent, concurrent, and divergent testing. Convergent validity was evidenced by strong alignment between the SMAS-7 and the LMAS-14, confirming conceptual consistency with an established adherence measure. Concurrent validity was supported by significant associations with the IFDFW scale and patient experience indices, underscoring the links between adherence, financial strain, and perceived quality of pharmaceutical care.^56^^,^^57^ Divergent validity was demonstrated through the absence of association with the EQ-VAS, a global quality-of-life measure conceptually distinct from adherence behavior.^58^ The scale also showed excellent criterion validity, accurately differentiating between higher and lower adherence groups, with performance exceeding that of comparable instruments.^59^^,^^60^ Nonetheless, these results should be interpreted cautiously, as they may reflect characteristics of this relatively homogeneous and highly educated sample. Future studies in diverse populations are needed to confirm the scale's external validity.

The proportion of participants demonstrating suboptimal adherence in this study closely aligns with global estimates, where nearly half of patients in developed settings are nonadherent,^61^^,^^62^ and is likely higher in lower-middle-income countries such as Lebanon.^63^ This pattern is concerning, as poor adherence contributes to therapeutic failure, increased morbidity, and greater healthcare expenditures.^64^ Several contextual factors in Lebanon may explain this trend. The ongoing economic crisis has severely reduced purchasing power and access to medications, even for essential chronic therapies.^65^ In addition, the fragmented healthcare system with limited coordination between public, private, and donor-driven entities, undermines continuity of care and patient trust.^66^ Cultural tendencies toward self-management and reliance on symptom improvement as a signal for recovery may also lead to premature treatment discontinuation, as reflected in several SMAS-7 items.^67^ Collectively, these factors indicate that suboptimal adherence is not merely a behavioral issue but a reflection of systemic, financial, and informational challenges requiring policy-level attention.

The analysis of predictors revealed a multifaceted picture of adherence behavior. Female participants reported lower adherence, consistent with literature suggesting that women may experience greater caregiving demands and competing health priorities that disrupt regular medication use.^68^ Regional differences, particularly lower adherence in Mount Lebanon and South Lebanon compared with Beirut, may stem from disparities in healthcare infrastructure and access to pharmacists.^69^ Financial well-being emerged as a strong determinant, reaffirming that economic stability directly influences the ability to sustain medication adherence.^70^

Clinical and experiential findings provided further insight. Individuals with chronic illnesses tended to be more adherent, a result often attributed to their stronger perceived need for medication, established therapeutic routines, and frequent healthcare interactions.^71^ Health coverage, whether through the National Social Security Fund or private insurance, also supported better adherence, illustrating the protective role of financial support mechanisms.^72^ Moreover, positive patient perceptions of pharmacist care promoted adherence, whereas communication barriers were associated with poorer outcomes, underscoring the central influence of pharmacist–patient relationships in a pharmacy-centered healthcare system such as Lebanon's.^73^^,^^74^

When these determinants were analyzed jointly, gender, region, financial well-being, chronic illness, and patient–pharmacist dynamics remained independent predictors of adherence. These findings highlight that interventions must be multidimensional: financial support alone is insufficient without parallel efforts to improve communication, patient education, and continuity of pharmaceutical care.

### 4.1 Practical implications

This study introduces a validated, brief, and multidimensional medication adherence scale that is applicable to both chronic and acute medication users in the general population. The SMAS-7 combines the brevity and usability of short scales with a conceptual richness that captures psychological, economic, and behavioral drivers of adherence. The scale is especially relevant for researchers and practitioners in low- and middle-income countries, where time and resources are limited but adherence remains critical for disease control.

In addition, the analysis provides relevant insights into adherence patterns, identifying modifiable and structural determinants that can inform policy, educational campaigns, and pharmacist training. Indeed, the findings underscore the need for multifaceted interventions that target financial barriers, improve patient-pharmacist communication, and enhance perceptions of care quality. The demonstrated influence of patient experience with pharmacy services suggests that adherence interventions should extend beyond the prescriber's office and into community-based care models.

From a clinical perspective, the SMAS-7 has potential for rapid assessment of medication adherence within community pharmacy and primary care settings, where time and resources are often constrained. Its brevity and multidimensional structure make it suitable for integration into medication reviews, counseling sessions, or digital adherence platforms. As a brief screening tool, SMAS-7 prioritizes feasibility, and thus may not fully encompass all adherence dimensions; longer or domain-specific instruments can be used alongside it when deeper profiling is required. Moreover, practical barriers should be acknowledged, including time limitations during consultations, variability in patient health literacy, and the need for potential language adaptation in multicultural contexts. Addressing these factors will be essential to ensure the scale's feasibility and consistent interpretation in routine care.

The present findings extend existing evidence by showing that medication adherence can be reliably assessed through a concise yet multidimensional tool applicable across both chronic and acute contexts. The SMAS-7 complements existing scales by integrating psychological, economic, and behavioral factors in a practical format suitable for clinical and population research. Its use can facilitate early identification of adherence barriers and inform targeted, multidimensional interventions, while future studies should confirm its validity in other clinical and cultural settings.

### 4.2 Limitations

Several limitations should be acknowledged. First, the cross-sectional design limits the ability to draw causal inferences regarding the relationships between adherence and its predictors. Second, the recruitment strategy using a snowball sampling approach and online dissemination through social media platforms may have introduced selection bias. Participants are likely to share similar social, educational, or professional networks, which could limit the diversity of the sample. Moreover, this digital approach may have underrepresented older adults, individuals with lower digital literacy, or those without stable internet access, thereby reducing the generalizability of the findings to these subgroups. Third, although the multivariable analyses adjusted for a wide range of predictors, residual confounding cannot be entirely ruled out, particularly in a complex context like Lebanon, where medication adherence may be influenced by fluctuating socioeconomic conditions, political and conflict-related instability. Lastly, because the primary objective was to validate the SMAS-7 in the general population, the scale was not stratified or tested across specific disease states, which limits insight into its performance in condition-specific contexts. Further research is warranted to address these limitations.

## 5 Conclusion

This study presents the development and initial psychometric validation of the SMAS-7, a concise, multidimensional tool for assessing medication adherence. The results provide promising evidence of reliability and construct validity within a national Lebanese sample. The scale demonstrated excellent validity, stability, and reliability, as well as strong criterion-related performance, offering a practical and robust alternative to longer existing instruments. The SMAS-7 represents a valuable resource for researchers, clinicians, and policymakers aiming to monitor and improve medication adherence in complex settings. However, further external and cross-cultural validations in clinical and international contexts are warranted before its broader adoption or use in clinical practice.

Nearly half of the population exhibited suboptimal adherence, underscoring the urgent need for scalable interventions. Several independent predictors of adherence were identified, highlighting the complex and multifactorial nature of medication adherence.

## CRediT authorship contribution statement

**Fouad Sakr:** Writing – review & editing, Writing – original draft, Visualization, Validation, Supervision, Software, Resources, Project administration, Methodology, Investigation, Formal analysis, Data curation, Conceptualization. **Mariam Dabbous:** Writing – review & editing, Writing – original draft. **Jihan Safwan:** Writing – review & editing. **Mohamad Rahal:** Writing – review & editing. **Pascale Salameh:** Writing – review & editing.

## Consent for publication

Not applicable.

## Ethics approval and consent to participate

The Ethics and Research Committee at the Lebanese International University approved the project (Approval number: 2025ERC-009-LIUSOP). Before enrolling in the survey, informed consent was obtained from all participants. Participation was voluntary, and respondents received no incentive in return for their participation.

## Funding

This research did not receive any specific grant from funding agencies in the public, commercial, or not-for-profit sectors.

## Declaration of competing interest

The authors have nothing to declare.

## Data Availability

The datasets generated during and/or analyzed during the current study are available from the corresponding author upon reasonable request.

## Appendix A Supplementary data

Supplementary material 1 Image 1

Supplementary material 2 Image 2

Supplementary material 3 Image 3

Supplementary material 4 Image 4

Supplementary material 5 Image 5
