# Supplementary material for: The short medication adherence scale (SMAS-7): Development and psychometric validation in a general population sample
Source: Explor Res Clin Soc Pharm. 2025 Oct 25;20:100676. doi: 10.1016/j.rcsop.2025.100676 (PMC12615316; doi:10.1016/j.rcsop.2025.100676)
Supplement: Supplementary file 1 — Supplementary material 1 [file mmc1.pdf]

# استبيان

عزيزي/ عزيزتي المشارك(ة)، أنت مدعو(ة) للمشاركة في هذه الدراسة التي تجريها مجموعة من الباحثين الأكاديميين في لبنان.

سيتم التعامل مع جميع المعلومات التي سيتم جمعها بسرية. مشاركتك في هذه الدراسة طوعية. يتطلب إكمال الاستبيان 15 دقيقة ويشير إلى موافقتك على المشاركة. شكرا لكم مقدما على وقتكم ومشاركتكم.

- ☐ لقد قرأت وفهمت المعلومات الواردة أعلاه.
- ☐ أفهم أن مشاركتي طوعية.
- ☐ أفهم أن بياناتي ستبقى سرية.
- ☐ أوافق على المشاركة في هذه الدراسة.

**القسم 1: الخصائص الاجتماعية والديموغرافية والاقتصادية**

1. العمر (بالسنوات): ____________

2. الجنس
   - ☐ ذكر
   - ☐ أنثى

3. عدد الأشخاص الذين يعيشون في نفس المنزل، بما فيهم أنت: ___________________

4. مكان الإقامة:
   - ☐ بيروت
   - ☐ البقاع
   - ☐ جبل لبنان
   - ☐ الشمال
   - ☐ الجنوب

5. الحالة الاجتماعية:
   - ☐ أعزب - عزباء
   - ☐ متزوج(ة)
   - ☐ مطلق(ة)
   - ☐ أرمل(ة)

6. المستوى التعليمي:
   - ☐ غير متعلم(ة)
   - ☐ مستوى مدرسي
   - ☐ مستوى جامعي

7. المهنة:
   - ☐ لا أعمل
   - ☐ موظف(ة)/أعمل لحسابي
   - ☐ متقاعد(ة)

8. دخل الأسرة الشهري:
   - ☐ أقل من 500$
   - ☐ 501$ – 999$
   - ☐ 1000$– 1500$
   - ☐ أكثر من 1500$

9. مقياس الضائقة المالية / الرفاهية المالية - IFDFW Scale في الأسابيع الأربعة الماضية

| الأسئلة | يرجى الإجابة برقم من 1 إلى 10 | | الرقم |
|---|---|---|---|
| ما هو شعورك حول مستوى ضغطك المالي اليوم؟ | 1= ضغط ساحق | 10= لا ضغط على الإطلاق | |
| ما مدى رضاك عن وضعك المالي الحالي؟ | 1= غير راضٍ/ية على الإطلاق | 10= راضٍ/ية | |
| ما هو شعورك حول وضعك المالي الحالي؟ | 1= أشعر بالعجز | 10= أشعر بالراحة | |
| كم مرة تقلق/ين بشأن قدرتك على تلبية نفقات المعيشة الشهرية العادية؟ | 1= قلق طوال الوقت | 10= لا قلق أبداً | |
| ما مدى ثقتك في أنه يمكنك العثور على المال لدفع ثمن أي طارئ مالي قد يكلف حوالي 5 مليون ل.ل. | 1= لا ثقة | 10= ثقة عالية | |
| كم مرة يحدث هذا لك: تريد/ين الخروج لتناول الطعام أو الذهاب إلى السينما أو القيام بنشاط آخر ولا تذهب/ين لأنك لا تستطيع/ين دفع الثمن؟ | 1= طوال الوقت | 10= أبداً | |
| كم مرة تجد/ين نفسك تحاول/ين أن تتدبر/ي أمرك مادياً لتعيش/ي بانتظار الأجر المقبل؟ | 1= طوال الوقت | 10= أبداً | |
| كيف هو شعورك بالضغط تجاه ماليّتك الشخصية بشكل عام؟ | 1= ضغط ساحق | 10= لا ضغط على الإطلاق | |

**القسم 2: الخصائص السريرية، بما في ذلك الحالة الصحية**

10. كيف تقيم(ي) حالتك الصحية؟
    - ☐ لا مرض
    - ☐ مرض مزمن

11. يرجى تحديد الحالات الطبية التي تعاني منها حالياً:

| | نعم | كلا |
|---|---|---|
| ضغط الدم | ☐ | ☐ |
| السكري | ☐ | ☐ |
| الكلسترول و الدهنيات | ☐ | ☐ |

| | | |
|---|---|---|
| أمراض القلب | ☐ | ☐ |
| أمراض الكلى | ☐ | ☐ |
| هل تعاني من الربو أو مرض الانسداد الرئوي المزمن أو غيره من الأمراض الرئوية المزمنة؟ | ☐ | ☐ |
| هل تعاني من حالات الحساسية؟ | ☐ | ☐ |
| هل لديك تاريخ من الجلطة الدماغية أو الأمراض الاوعية الدماغية؟ | ☐ | ☐ |
| هل لديك نوبات صرع أو حالات عصبية أخرى؟ | ☐ | ☐ |
| هل تعاني من الاكتئاب أو القلق أو حالات نفسية أخرى؟ | ☐ | ☐ |
| هل لديك أي أمراض في المعدة أو الامعاء؟ | ☐ | ☐ |
| هل لديك أي سرطان حالي أو سابق؟ | ☐ | ☐ |
| هل تعاني من أمراض الروماتيزم؟ | ☐ | ☐ |
| هل لديك أي أمراض مزمنة أخرى؟ | ☐ | ☐ |

12. إذا كانت الإجابة نعم، كم عدد الأمراض المزمنة الأخرى التي تعاني منها؟ (ضع صفر إذا لم يكن هناك مرض مزمن)

13. ما عدد الأدوية التي تتناولها بشكل روتيني أو منتظم يوميا؟ (ضع صفر إذا لا ينطبق)

14. هل لديك وصول سهل إلى الرعاية الصحية؟
☐ لا
☐ نعم

15. ما هي التغطية الصحية الخاصة بك؟
☐ تأمين خاص
☐ الصندوق الوطني للضمان الاجتماعي
☐ تأمين القطاع العام
☐ لا تغطية صحية

16. مقياس الالتزام بالأدوية اللبناني (LMAS-14)

| **الرجاء الإجابة على الأسئلة التالية بإختيار الرقم المناسب لكل سؤال من 1 (التزام أقل) إلى 4 (التزام أعلى)** | | | | |
|---|---|---|---|---|
| | **1** | **2** | **3** | **4** |
| 1. هل تنسى تناول دواءك عندما تكون مشغولاً (العمل المكثف أو السفر)؟ | | | | |
| 2. هل تنسى تناول دواءك إذا كنت مدعو لتناول الغداء / العشاء؟ | | | | |
| 3. هل تنسى تناول دواءك؟ | | | | |
| 4. هل تتأخر عندما يتعلق الأمر بشراء عبوات دواءك عندما تنفذ؟ | | | | |
| 5. هل تتوقف عن تناول الدواء إذا كان يمنعك من تناول بعض الأطعمة التي تحبها بسبب التفاعل المحتمل بين الدواء والأكل؟ | | | | |
| 6. هل ستتوقف عن تناول دواءك دون استشارة طبيبك إذا أخذ قريبك / جارك الدواء نفسه لفترة طويلة وتسبب ذلك في آثار جانبية؟ | | | | |
| 7. هل ستتوقف عن تناول دواءك دون استشارة طبيبك أظهرت الفحوصات المخبرية تحسناً خلال فترة العلاج؟ | | | | |

| | | | | |
|---|---|---|---|---|
| 8. هل تتوقف عن تناول دواءك دون استشارة طبيبك إذا لم تشعر بتحسن خلال فترة العلاج؟ | | | | |
| 9. هل تتوقف عن تناول دواءك دون استشارة طبيبك إذا شعرت بتحسن خلال فترة العلاج؟ | | | | |
| 10. هل تقرر إيقاف بعض أدويتك دون استشارة طبيبك إذا لاحظت أنك تتناول الكثير من الأدوية كل يوم؟ | | | | |
| 11. هل تتوقف عن أخذ علاجك المزمن إذا شعرت بالملل منه؟ | | | | |
| 12. هل تتوقف عن تناول دواءك في حال حصول آثار جانبية؟ | | | | |
| 13. هل تتوقف عن تناول دواءك إذا كان تأمينك لا يغطيه؟ | | | | |
| 14. هل تتوقف عن شراء أدويتك إذا كنت تعتبرها غالية؟ | | | | |

## القسم 3: تقييم العلاقة بين الصيدلي والمريض

17. هل تتلقى استشارات منتظمة من الصيدلي؟
- ☐ لا على الإطلاق
- ☐ نعم ، بانتظام
- ☐ نعم ، من وقت لآخر

18. إذا تلقيت استشارة من الصيدلي ، فكم من الوقت يقضيه الصيدلي في تقديم المشورة لك بشأن دواء و / أو حالة طبية؟
- ☐ أقل من 5 دقائق
- ☐ من 5 إلى 10 دقائق
- ☐ أكثر من 10 دقائق

19. مؤشر توقعات المريض:

| **يرجى الإجابة ب "نعم" أو "لا" على كل من العبارات التالية.**<br>**أنا أعتبر خدمة الصيدلية جيدة في حال:** | | |
|---|---|---|
| | **نعم** | **لا** |
| 1. توافر الصيدلي في جميع الأوقات في الصيدلية | ☐ | ☐ |
| 2. استجابة سريعة للأسئلة | ☐ | ☐ |
| 3. خدمة سريعة | ☐ | ☐ |
| 4. الاحترام | ☐ | ☐ |
| 5. التعاطف | ☐ | ☐ |
| 6. الاستشارة الجيدة | ☐ | ☐ |
| 7. توافر أي منتج صيدلاني مطلوب | ☐ | ☐ |
| 8. تقديم الخدمات (قياس ضغط الدم ، قياس السكري ، إلخ) | ☐ | ☐ |
| 9. تقديم عينات تجميلية | ☐ | ☐ |
| 10. أسعار وخصومات أفضل | ☐ | ☐ |
| 11. التوصيل أو الخدمات المنزلية | ☐ | ☐ |

20. عوائق التواصل مع صيدلي المجتمع:

| يرجى الإجابة ب "نعم" أو "لا" كل من العبارات التالية: | | |
|---|---|---|
| | نعم | لا |
| 1. لا خصوصية في الصيدلية | ☐ | ☐ |
| 2. لا أثق بالصيدلي | ☐ | ☐ |
| 3. السلوك السلبي للصيدلي (الوقاحة وعدم الود) | ☐ | ☐ |
| 4. الخوف من التهويل **على** | ☐ | ☐ |
| 5. نقص المعرفة الصيدلانية | ☐ | ☐ |
| 6. الصيدلي ليس لديه وقت لتقديم المشورة | ☐ | ☐ |
| 7. الصيدلي غير متوفر دائما في الصيدلية | ☐ | ☐ |

21. مؤشر إدراك المريض:

| يرجى الإشارة إلى مستوى موافقتك على كل مما يلي من العبارات التالية حول دور صيدلي المجتمع.<br>دور الصيدلي في الصيدلية هو: | | | |
|---|---|---|---|
| | لا أوافق | محايد / لا أعرف | أوافق |
| 1. تقديم المشورة بشأن استخدام الادوية وتناولها | ☐ | ☐ | ☐ |
| 2. التحقق من التفاعلات الدوائية | ☐ | ☐ | ☐ |
| 3. تقديم المشورة بشأن التفاعلات بين الادوية والاغذية | ☐ | ☐ | ☐ |
| 4. تقديم المشورة حول المرض وكيفية علاجه | ☐ | ☐ | ☐ |
| 5. تشخيص مشكلتي الصحية وإعطائي العلاج | ☐ | ☐ | ☐ |
| 6. التحقق من دقة الوصفة الطبية الخاصة بي فيما يتعلق باسم الدواء والجرعة | ☐ | ☐ | ☐ |
| 7. تقديم المشورة بشأن الآثار الجانبية للأدوية | ☐ | ☐ | ☐ |
| 8. إخباري متى يجب الذهاب إلى طبيب أو إلى المستشفى | ☐ | ☐ | ☐ |
| 9. إعطائي نصائح غير دوائية | ☐ | ☐ | ☐ |
| 10. الاستماع إلى مشاكل المرضى | ☐ | ☐ | ☐ |
| 11. متابعة المرضى | ☐ | ☐ | ☐ |
| 12. القدرة على إعطاء الحقن عند الحاجة | ☐ | ☐ | ☐ |
| 13. القدرة على تفسير نتائج الفحوصات الخاصة بي | ☐ | ☐ | ☐ |
| 14. صرف الدواء الصحيح فقط | ☐ | ☐ | ☐ |

**القسم 4: تقييم رضا المرضى عن الصيادلة والخدمات المجتمعية**

22. النموذج القصير المعدل لاستبيان رضا المريض (MA-PSQ-18)

| يرجى الإشارة إلى مدى موافقتك أو عدم موافقتك على كل من العبارات التالية. | | | | | |
|---|---|---|---|---|---|
| | أوافق بشدة | أوافق | غير متأكد | لا أوافق | لا أوافق بشدة |
| 1. الصيادلة جيدون في شرح سبب الفحوصات الطبية | ☐ | ☐ | ☐ | ☐ | ☐ |
| 2. أعتقد أن الصيدلية التي أزورها بانتظام لديها كل ما يلزم لتوفير الرعاية الطبية الكاملة | ☐ | ☐ | ☐ | ☐ | ☐ |

| | | | | | |
|---|---|---|---|---|---|
| 3. الرعاية الطبية التي أتلقاها من قبل الصيدلي مثالية تقريباً | ☐ | ☐ | ☐ | ☐ | ☐ |
| 4. الصيادلة لا يجعلوني أتساءل عما إذا كانت الرعاية الطبية المقدمة منهم صحيحة | ☐ | ☐ | ☐ | ☐ | ☐ |
| 5. أشعر بالثقة في أنني أستطيع الحصول على الرعاية الطبية التي أحتاجها من الصيدلية التي أزورها بانتظام دون أن أتعرض لانتكاسة مالية | ☐ | ☐ | ☐ | ☐ | ☐ |
| 6. عندما أذهب إلى الصيدلية، فإنهم حريصون على التحقق من كل شيء عند تقديم الرعاية الطبية لي | ☐ | ☐ | ☐ | ☐ | ☐ |
| 7. لست مضطراً لدفع أكثر مما أستطيع تحمله مقابل رعايتي الطبية في الصيدلية | ☐ | ☐ | ☐ | ☐ | ☐ |
| 8. لدي سهولة الوصول إلى الصيادلة الأكفاء حسب حاجتي | ☐ | ☐ | ☐ | ☐ | ☐ |
| 9. حيثما أحصل على الرعاية الصيدلانية، لا يضطر الناس إلى الانتظار طويلا للعلاج في حالات الطوارئ | ☐ | ☐ | ☐ | ☐ | ☐ |
| 10. الصيادلة ليسوا تجار وغير مبالين تجاهي | ☐ | ☐ | ☐ | ☐ | ☐ |
| 11. يعتني بي الصيادلة بطريقة ودية ومهذبة للغاية | ☐ | ☐ | ☐ | ☐ | ☐ |
| 12. الصيادلة الذين يقدمون لي الرعاية الطبية ليسوا في عجلة من أمرهم عندما يقدمون الرعاية لي | ☐ | ☐ | ☐ | ☐ | ☐ |
| 13. الصيادلة لا يتجاهلون ما أقوله لهم | ☐ | ☐ | ☐ | ☐ | ☐ |
| 14. أثق في قدرة الصيادلة الذين يقدمون لي الرعاية | ☐ | ☐ | ☐ | ☐ | ☐ |
| 15. عادة يقضي الصيادلة وقتاً كافياً معي | ☐ | ☐ | ☐ | ☐ | ☐ |
| 16. من السهل الحصول على الرعاية الطبية فوراً في الصيدلية | ☐ | ☐ | ☐ | ☐ | ☐ |
| 17. أنا راضي عن الرعاية الطبية التي أتلقاها من قبل الصيادلة | ☐ | ☐ | ☐ | ☐ | ☐ |
| 18. أنا قادر على الحصول على الرعاية الطبية من الصيادلة كلما احتجت إليها | ☐ | ☐ | ☐ | ☐ | ☐ |

**القسم 5: تقييم التزام المرضى بعلاجاتهم.**

23. مقياس الالتزام الدوائي القصير (SMAS-7).

| **الرجاء الإجابة على الأسئلة التالية بإختيار الرقم المناسب لكل سؤال من 1 (التزام أقل) إلى 4 (التزام أعلى)** | | | | |
|---|---|---|---|---|
| | **1** | **2** | **3** | **4** |
| 1. هل تنسى تناول دواءك؟ | | | | |
| 2. هل تتأخر عندما يتعلق الأمر بشراء عبوات دواءك عندما تنفذ؟ | | | | |
| 3. هل ستتوقف عن تناول دواءك دون استشارة طبيبك أظهرت الفحوصات المخبرية تحسناً خلال فترة العلاج؟ | | | | |
| 4. هل تتوقف عن تناول دواءك دون استشارة طبيبك إذا لم تشعر بتحسن خلال فترة العلاج؟ | | | | |
| 5. هل تتوقف عن تناول دواءك دون استشارة طبيبك إذا شعرت بتحسن خلال فترة العلاج؟ | | | | |
| 6. هل تتوقف عن تناول دواءك إذا كان تأمينك لا يغطيه؟ | | | | |
| 7. هل تتوقف عن شراء أدويتك إذا كنت تعتبرها غالية؟ | | | | |

**القسم 6: استبيان الحالة الصحية.**

24. استبيان EQ-5D-5L لجودة الحياة.

| | |
|---|---|
| أنقر من فضلك مربعًا **واحدًا** لتشير إلى أفضل عبارة تصف حالتك الصحية **اليوم**. | |
| **القدرة على الحركة** | |
| ليس لدي أي مشاكل أثناء المشي | ☐ |
| أعاني مشاكل طفيفة أثناء المشي | ☐ |
| أعاني مشاكلَ متوسطة أثناء المشي | ☐ |
| أعاني مشاكلَ حادّة أثناء المشي | ☐ |
| ليس لدي القدرة على المشي | ☐ |
| **العناية الشخصية** | |
| ليس لدي أي مشاكل في الاستحمام أو ارتداء ملابسي بنفسي | ☐ |
| أعاني مشاكلَ طفيفة عند الاستحمام أو ارتداء ملابسي بنفسي | ☐ |
| أعاني مشاكلَ متوسطة عند الاستحمام أو ارتداء ملابسي بنفسي | ☐ |
| أعاني مشاكلَ حادّة عند الاستحمام أو ارتداء ملابسي بنفسي | ☐ |
| ليس لدي القدرة على الاستحمام أو ارتداء الملابس بنفسي | ☐ |
| **الأنشطة المعتادة (مثل العمل، الدراسة، الأعمال المنزلية، الأنشطة الأسرية أو الترفيهية)** | |
| ليس لدي أي مشاكل في ممارسة نشاطاتي المعتادة | ☐ |
| أعاني مشاكلَ طفيفة في القيام بنشاطاتي المعتادة | ☐ |
| أعاني مشاكلَ متوسطة في ممارسة نشاطاتي المعتادة | ☐ |
| أعاني مشاكلَ حادّة في ممارسة نشاطاتي المعتادة | ☐ |
| ليس لديّ القدرة على ممارسة نشاطاتي المعتادة | ☐ |
| **الألم / الإحساس بعدم الراحة** | |
| ليس لدي أي ألم أو انزعاج | ☐ |
| أعاني ألماً طفيفاً أو انزعاجاً طفيفاً | ☐ |
| أعاني ألماً متوسطاً أو انزعاجاً متوسطاً | ☐ |
| أعاني ألماً حادّاً أو انزعاجاً حادّاً | ☐ |
| أعاني ألماً شديداً جداً أو انزعاجاً شديداً جداً | ☐ |
| **القلق / الاكتئاب** | |
| لا أعاني أيّ قلق أو اكتئاب | ☐ |
| أعاني قلقاً طفيفاً أو اكتئاباً طفيفاً | ☐ |
| أعاني قلقاً متوسطاً أو اكتئاباً متوسطاً | ☐ |
| أعاني قلقاً حادّاً أو اكتئاباً حادّاً | ☐ |
| أعاني قلقاً شديداً جداً أو اكتئاباً شديداً جداً | ☐ |
| نود أن نعرف مدى سوء حالتك الصحية أو سلامتها **اليوم**.<br>هذا المقياس مدرج من الرقم **0 إلى 100**.<br>الرقم 100 يعني <u>أحسن</u> حالة صحية يمكنك تصوّرها.<br>0 يعني <u>أسوأ</u> حالة صحية يمكنك تصوّرها.<br>يرجى كتابة الرقم الذي يشير إلى صحتك <u>اليوم</u>. | |
| **حالتك الصحية اليوم =** ____________________ | |
