# Supplementary material for: The short medication adherence scale (SMAS-7): Development and psychometric validation in a general population sample
Source: Explor Res Clin Soc Pharm. 2025 Oct 25;20:100676. doi: 10.1016/j.rcsop.2025.100676 (PMC12615316; doi:10.1016/j.rcsop.2025.100676)
Supplement: Supplementary file 2 — Supplementary material 2 [file mmc2.pdf]

Supplementary Table 1: Sociodemographic and socioeconomic characteristics of the participants

Variable	Mean / Frequency	SD / %
**Age (years)**	34.66	15.17
**Gender**		
Male	164	32.73 %
Female	337	67.27 %
**Region**		
Beirut	115	22.95 %
Bekaa	10	2.00 %
Mount Lebanon	15	2.99 %
North	18	3.59 %
South	343	68.46 %
**Marital status**		
Single	262	52.30 %
Married	216	43.11 %
Divorced / Widowed	23	4.59 %
**Education level**		
Not educated	12	2.40 %
School level	80	15.97 %
University level	409	81.64 %
**Occupation**		
Unemployed	202	40.32 %
Employed / Self-employed	283	56.49 %
Retired	16	3.19 %
**Monthly income (USD)**		
< 500	110	21.96 %
501 – 999	112	22.36 %
1 000 – 1500	119	23.75 %
> 1500	160	31.94 %
**Total IFDFW score**	47.94	19.92

SD: standard deviation; USD: US Dollars; IFDFW: InCharge Financial Distress/Financial Well-Being Scale.
