# Supplementary material for: The short medication adherence scale (SMAS-7): Development and psychometric validation in a general population sample
Source: Explor Res Clin Soc Pharm. 2025 Oct 25;20:100676. doi: 10.1016/j.rcsop.2025.100676 (PMC12615316; doi:10.1016/j.rcsop.2025.100676)
Supplement: Supplementary file 3 — Supplementary material 3 [file mmc3.pdf]

Supplementary Table 2: Clinical characteristics and patient-reported experiences

Variable	Mean / Frequency	SD / %
**Current health status**		
No chronic illness	371	74.05 %
Chronic illness	130	25.95 %
**Total number of comorbidities**	1.33	1.56
**Number of chronic daily medications**	1.04	1.66
**Easy access to healthcare**		
No	101	20.16 %
Yes	400	79.84 %
**Health coverage**		
No coverage	192	38.32 %
National Social Security Fund (NSSF)	84	16.77 %
Public insurance	57	11.38 %
Private insurance	168	33.53 %
**Do you receive regular counseling by a pharmacist?**		
No, not at all	128	25.55 %
Yes, from time to time	258	51.50 %
Yes, regularly	115	22.95 %
**Duration of counseling by a pharmacist, once received**		
Less than 5 minutes	221	44.11 %
5 to 10 minutes	236	47.11 %
More than 10 minutes	44	8.78 %
**Patient Expectation Index score**	20.09	2.01
**Barriers for Communication with Pharmacist score**	9.69	2.70
**Patient Perception Index score**	35.22	6.51
**MA-PSQ18 score**	69.35	10.75
**LMAS-14 score**	36.66	11.04
**EQ VAS score**	76.16	20.82

SD: standard deviation; MA-PSQ18: Modified Arabic version of the Patient Satisfaction Questionnaire Short Form; LMAS-14: 14-item Lebanese Medication Adherence Scale.
