# Supplementary material for: The short medication adherence scale (SMAS-7): Development and psychometric validation in a general population sample
Source: Explor Res Clin Soc Pharm. 2025 Oct 25;20:100676. doi: 10.1016/j.rcsop.2025.100676 (PMC12615316; doi:10.1016/j.rcsop.2025.100676)
Supplement: Supplementary file 4 — Supplementary material 4 [file mmc4.pdf]

Supplementary Table 3: Bivariate Pearson correlation among the SMAS-7 and related study variables.

Validity type	Variables correlated with SMAS-7	r	P-value
**Convergent validity**	LMAS-14	0.974	< 0.001
**Concurrent validity**	IFDFW	0.139	0.002
	Patient Perception Index	0.117	0.009
	MA-PSQ-18	0.089	0.047
**Divergent validity**	EQ VAS	0.072	0.108

r = Pearson correlation coefficient.
