# Supplementary material for: The short medication adherence scale (SMAS-7): Development and psychometric validation in a general population sample
Source: Explor Res Clin Soc Pharm. 2025 Oct 25;20:100676. doi: 10.1016/j.rcsop.2025.100676 (PMC12615316; doi:10.1016/j.rcsop.2025.100676)
Supplement: Supplementary file 5 — Supplementary material 5 [file mmc5.pdf]

Supplementary Table 4: Univariate logistic regression analysis of sociodemographic, socioeconomic, clinical and patient-reported experience factors associated with the dichotomized SMAS-7 score

Variable	Unadjusted OR	95% CI Lower	95% CI Upper	P value
**Age**	1.020	1.008	1.032	0.001
**Gender**				
Female vs. Male	0.449	0.306	0.656	< 0.001
**Region** (reference: Beirut)				
Bekaa	1.152	0.302	5.571	0.844
Mount Lebanon	0.179	0.047	0.563	0.005
North	0.395	0.140	1.080	0.071
South	0.353	0.225	0.547	< 0.001
**Marital status** (reference: Single)				
Married	1.563	1.089	2.249	0.016
Divorced/ Widowed	0.568	0.212	1.378	0.229
**Education level** (reference: Not educated)				
School level	1.721	0.499	6.874	0.405
University level	1.877	0.582	7.121	0.310
**Occupation** (reference: Unemployed)				
Employed/ Self-employed	1.100	0.766	1.580	0.606
Retired	1.507	0.541	4.365	0.433
**Monthly income** (reference: Less than 500 USD)				
501 - 999 USD	0.935	0.539	1.621	0.810
1000 - 1500 USD	1.969	1.163	3.361	0.012
More than 1500 USD	2.694	1.640	4.477	< 0.001
**Total IFDFW score**	1.019	1.009	1.028	< 0.001
**Current health status**				
Chronic illness vs No illness	2.329	1.549	3.532	< 0.001
**Total number of comorbidities**	0.964	0.858	1.078	0.521
**Number of chronic daily medications**	1.022	0.919	1.137	0.690
**Easy access to healthcare**				
Yes vs. No	1.510	0.972	2.367	0.069
**Health coverage** (reference: No coverage)				
National Social Security Fund (NSSF)	2.026	1.208	3.422	0.008
Public insurance	1.246	0.681	2.263	0.472
Private insurance	1.977	1.301	3.020	0.001
**Do you receive regular counseling by a pharmacist?** (reference: No, not at all)				
Yes, from time to time	1.001	0.655	1.535	0.995
Yes, regularly	1.515	0.914	2.521	0.108
**Duration of counseling by a pharmacist, once received** (reference: Less than 5 minutes)				
5 to 10 minutes	1.150	0.796	1.662	0.456

More than 10 minutes	1.452	0.759	2.804	0.261
**Patient Expectation Index score**	0.946	0.866	1.032	0.215
**Barriers for Communication with Pharmacist score**	0.884	0.827	0.945	< 0.001
**Patient Perception Index score**	1.060	1.030	1.092	< 0.001
**MA-PSQ18 score**	1.027	1.010	1.044	0.002
**EQ VAS score**	1.005	0.996	1.013	0.284

OR: odds ratio; CI: confidence interval; USD: US Dollars; IFDFW: InCharge Financial Distress/Financial Well-Being Scale; MA-PSQ18: Modified Arabic version of the Patient Satisfaction Questionnaire Short Form.
